# CCR6 expression reduces mouse survival upon malarial challenge with *Plasmodium berghei* NK65 strain

**DOI:** 10.1590/0074-02760210287

**Published:** 2022-06-17

**Authors:** Eduardo Lani Volpe da Silveira, Urvashi Rai, Vivian Bonezi, Carlos Rodrigo Zárate-Bladés, Carla Claser

**Affiliations:** 1 New York University School of Medicine, Department of Pathology, Michael Heidelberg Division of Immunology, New York, NY, United States of America; 2 Universidade de São Paulo, Faculdade de Ciências Farmacêuticas, Departamento de Análises Clínicas e Toxicológicas, Laboratório de Imunologia de Células B, São Paulo, SP, Brasil; 3 Universidade Federal de Santa Catarina, Departamento de Microbiologia, Imunologia e Parasitologia, Laboratório de Imunorregulação, Florianópolis, SC, Brasil; 4 Universidade de São Paulo, Instituto de Ciências Biomédicas, Departamento de Parasitologia, São Paulo, SP, Brasil

**Keywords:** malaria, sporozoites, iRBCs, chemokine receptor, Th1 cytokines

## Abstract

**BACKGROUND:**

It has been demonstrated that proteins expressed by liver-stage *Plasmodium* parasites can inhibit the translocation of transcription factors to the nucleus of different cells. This process would hinder the expression of immune genes, such as the CCL20 chemokine.

**OBJECTIVE:**

Since CCR6 is the only cognate receptor for CCL20, we investigated the importance of this chemokine-receptor axis against rodent malaria.

**METHODS:**

CCR6-deficient (KO) and wild-type (WT) C57BL/6 mice were challenged with *Plasmodium berghei* (Pb) NK65 sporozoites or infected red blood cells (iRBCs). Liver parasitic cDNA, parasitemia and serum cytokine concentrations were respectively evaluated through reverse transcription-polymerase chain reaction (RT-PCR), staining thin-blood smears with Giemsa solution, and enzyme-linked immunosorbent assay (ELISA).

**FINDINGS:**

Although the sporozoite challenges yielded similar liver parasitic cDNA and parasitemia, KO mice presented a prolonged survival than WT mice. After iRBC challenges, KO mice kept displaying higher survival rates as well as a decreased IL-12 p70 concentration in the serum than WT mice.

**CONCLUSION:**

Our data suggest that malaria triggered by PbNK65 liver- or blood-stage forms elicit a pro-inflammatory environment that culminates with a decreased survival of infected C57BL/6 mice.

Malaria is a parasitic disease caused by multiple *Plasmodium* species that show distinct distributions worldwide and represents one of the largest global public health issues. In 2019, nearly 229 million cases and 409 thousand malaria-derived deaths were reported.^1^ Despite the efficiency of the current therapies, these high numbers of cases and mortality have been associated with the selection of drug-resistant parasites.^2^ Since the first malaria vaccine has been recently approved,^3^ there is a belief that a wide vaccination coverage may control this massive epidemiology impact in a midterm period.

*Plasmodium* infective forms are mosquito-transmitted sporozoites that majorly express the circumsporozoite protein (CSP) on their surface. After invading the host skin, these parasites migrate through the circulation until reaching the liver. Inside hepatocytes, they proliferate and differentiate into exo-erythrocytic forms (EEFs), finalizing the pre-erythrocytic stage of the infection. Of note, the CSP presence in the hepatocyte cytoplasm inhibits the functionality of the transcriptional factor NF-κB, reducing the gene expression related to immunity. Indeed, the transcriptome data from EEF-infected HepG2 cell cultures suggest that the CSP down-regulates in almost 65-fold the expression of the CCL20 (also named MIP-3α) chemokine gene.^4^

The only receptor known as capable of binding CCL20 is CCR6.^5^ The blood RNA consensus dataset from the human protein atlas (https://www.proteinatlas.org/ENSG00000112486-CCR6/blood) indicates that CCR6-expressing cells are mainly composed of T cells (regulatory, MAIT, and memory), B cells (naive and memory), NK cells, and dendritic cells (myeloid and plasmacytoid). Also, a DNA vaccination study performed with a CSP sequence fused with the CCL20 counterpart provided protection against a malarial challenge in mice^6^ and infant macaques.^7^ Considering the importance of all these cell CCR6+ subsets in the malaria immunity^8^ ^,^ ^9^ ^,^ ^10^ ^,^ ^11^ and the early lethality presented by C57BL/6 wild-type (WT) mice challenged with *Plasmodium berghei* NK65 (PbNK65),^9^ ^,^ ^12^ ^,^ ^13^ ^,^ ^14^ ^,^ ^15^ ^,^ ^16^ ^,^ ^17^ ^,^ ^18^ we hypothesized that CCR6 KO mice could be even lesser resistant than WT mice to this parasite.

## MATERIALS AND METHODS

*Mice* - Eight to ten-week-old female C57BL/6 mice were purchased from Taconic Farms Inc (USA). CCR6-deficient (CCR6 KO) mice from the same genetic background as C57BL/6 mice were kindly provided by Dr. Sergio Lira (Icahn School of Medicine at Mount Sinai, New York, USA). All animals were kept under specific pathogen-free conditions at the New York University LARC animal facility. The experiments were performed in accordance with the guidelines approved by the ethics committee of the New York University.

*Parasitic infection* - Mice were infected with either sporozoites or blood-stage PbNK65 strain parasites derived from the Department of Parasitology at the New York University (USA).^19^ ^,^ ^20^ ^,^ ^21^ For the liver-stage infections, sporozoite challenges were performed through two distinct methods: (a) intravenous (iv) injection of parasites obtained from salivary glands of infected *Anopheles stephensi* mosquitoes; (b) natural infection by mosquito bites. Eighteen days before the mouse infection, reared mosquitoes were infected with *Plasmodium bergh*ei NK65 upon a C57BL/6 mouse blood meal in the insectary from the Department of Parasitology at the New York University, USA) as previously described.^22^ On day 0, mice were anesthetized with ketamine and xylazine and exposed to the bites of PbNK65 strain-infected mosquitoes for 15 minutes. To ensure the infectivity of the mosquito bites, sporozoites harvested from the salivary glands of other mosquitoes from the same buckets were quantified before the challenge. Blood-stage challenges were performed with infected RBCs (iRBCs) harvested from a donor PbNK65-infected C57BL/6 mouse. More specifically, donor blood samples were collected at any time from day 4 to 10 post-infection, which corresponds to ascending periods of parasitemia and eventually stored at -80ºC until usage. Once thawed, parasites could be cycling at different stages, and iRBCs were resuspended in 1X RPMI 1640 medium and washed twice with the same culture medium solution through 600 x g centrifugation at 4ºC for 5 min. To measure the iRBC percentage in the donor mouse, a total of 40 fields were analyzed from each thin blood smear stained with Giemsa solution. Then, another batch of animals was intravenously injected with either 2,000 or 20,000 iRBCs.

*Enzyme-linked immunosorbent assay (ELISA)* - IL-12p70 and IFN-γ serum levels were measured through ELISA kits (Thermo-fisher 88-7121 and 88-7314, respectively), kindly provided by Dr. Moriya Tsuji (The Aaron Diamond AIDS Research Center - affiliated with The Rockefeller University, New York, USA). Serum samples from naïve and infected mice were collected on days 2, 4, and 6 post iRBC challenge and diluted 1:10 for these quantifications.

*Quantitative polymerase chain reaction (qPCR)* - Forty-two hours following a sporozoite challenge (iv injection or exposure to infected mosquito bites), mouse livers were excised and processed for the isolation of total RNA. Four micrograms of total RNA were used in the reverse transcription as previously described.^23^ The resulting cDNA was used as a template for a quantitative real-time PCR for Pb 18S rRNA sequences combined with the Pb 18S forward (5’ - AAGCATTAAATAAAGCGAATACATCCTTAC - 3’) and reverse (5’ - GGAGATTGGTTTTGACGTTTATGTG - 3’) primers. The amplification was performed with the iCycler iQ Real-Time PCR Detection System (Bio-Rad, Hercules, CA). The quantification of Pb18S normalized gene expression was based on the cycle threshold (Ct) values as described earlier.^23^ A double-stranded-DNA-specific iQ SYBR Green supermix (Biorad Laboratories) was used to detect the PCR products. As a positive control, a defined number of copies of a Pb18S rRNA plasmid standard was utilized.

*Statistical analysis* - Survival curves (Kaplan-Meier) were assessed using the log-rank Mantel-Cox test (Prisma Software). Two-independent sample student’s t-test (http://faculty.vassar.edu/lowry/VassarStats.htm) was used to compare the cytokine production between WT and CCR6 KO mice. A p-value < 0.05 indicated significant differences between the mouse strains.

## RESULTS

**Fig. 1:**
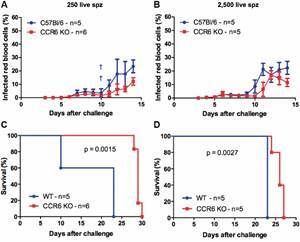
CCR6 KO mice displayed an extended survival upon a *Plasmodium berghei* NK65 sporozoite challenge in comparison to wild-type (WT) mice. Mice were infected via intravenous injection with an inoculum size similar to the parasite amount naturally transmitted by mosquitoes ((A) 250 or (B) 2,500 PbNK65 strain live sporozoites). A-B) Parasitemia levels (mean ± standard deviation) were quantified from three days after the challenge onward in Giemsa stained-blood smears slides. † Number of mice found dead. C-D) Kaplan-Meier curves represent the daily survival of challenged mice (C) 250 or (D) 2,500 PbNK65 strain live sporozoites). Log-rank test was performed on this analysis. Circles - WT mice; Squares - CCR6 KO mice. This experiment was performed only once.

*CCR6 expression diminishes malaria survival in PbNK65 sporozoite-infected mice* - To address the impact of the CCR6 expression in the immunity against PbNK65 malaria, our first approach was to challenge WT and CCR6 KO mice with different sporozoite numbers via iv injection. Thus, mice were infected with lethal inoculums ranging from 250 to 250,000 live parasites and parasitemia levels and survival were measured. In all sporozoite challenges, we found similar parasitemia levels among these mouse strains [Fig. 1A-B, Supplementary data (Fig. 1A-B)]. Surprisingly, CCR6 KO mice presented a significant delay in the mortality relative to WT mice when challenged with the smaller parasitic inoculums (250 sporozoites - p = 0.0015; 2,500 sporozoites - p = 0.0027) (Fig. 1C-D). However, this survival pattern observed in CCR6 KO mice disappeared with the challenges with 25,000 and 250,000 sporozoites (p = 0.0644 and p = 0.3173, respectively) [Supplementary data (Fig. 1C-D)].

**Fig. 2:**
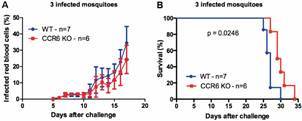
CCR6 KO mice displayed an extended survival against malaria transmitted by the bites of *Plasmodium berghei* NK65-infected mosquitoes relative to wild-type (WT) mice. Each mouse was anesthetized and exposed to the bites of three infected mosquitoes for 15 minutes. About 5,333 sporozoites were found in the salivary glands of other mosquitoes not used in the challenge but from the same bucket. (A) Parasitemia levels (mean ± standard deviation) were quantified from five days after the challenge onward in Giemsa stained-blood smears slides. (B) Kaplan-Meier curves represent the daily survival of challenged mice. Log-rank test was performed on this analysis. Circles - WT mice; Squares - CCR6 KO mice. This experiment was performed only once.

Another approach to evaluate the resistance of these animals against liver-stage PbNK65 parasites was to expose them to the bites of PbNK65-infected mosquitoes. Thus, a total of three, seven, or 15 infectious mosquitoes were allowed to bite WT and CCR6 KO mice for 15 min. To estimate how many sporozoites those mosquitoes had in their salivary glands, we determined the parasitic loads derived from the salivary glands of other mosquitoes not used in the challenges, but derived from the same buckets. They ranged from 5,333 to 14,000 sporozoites per mosquito. Regardless of the mosquito numbers used in this challenge, parasitemia levels remained equivalent among these mouse strains [Fig. 2A, Supplementary data (Fig. 2A-B)]. Similar to the previous sporozoite challenge via iv injection, CCR6 KO mice still displayed a prolonged survival when exposed to the bites of three infectious mosquitoes (p = 0.0246) (Fig. 2B). In contrast, the sporozoite challenges through the biting of seven or 15 infectious mosquitoes induced similar survival rates between WT and CCR6 mice (p = 0.0849 and p = 0.0639, respectively) [Supplementary data (Fig. 2C-D)].

**Fig. 3:**
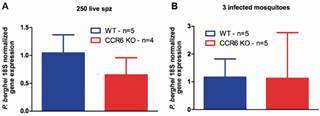
extended survival of CCR6 KO mice against *Plasmodium berghei* malaria induced by a live sporozoite injection or the exposure to infected-mosquito bites is independent of the pre-erythrocytic stage of the infection. Mice were infected via intravenous injection with 250 live PbNK65 sporozoites (A) or exposed to the bites of three infected mosquitoes (B). Forty-two hours after the challenge, mice were euthanized and livers were harvested for RNA extraction. To estimate the parasitic loads in the liver, the ensuing cDNA was analyzed for the normalized expression of Pb rRNA 18S through quantitative real-time PCR. GAPDH gene expression was utilized as a positive control. Data represent the mean ± standard deviation. Blue columns - wild-type (WT) mice; Red columns - CCR6 KO mice. This experiment was performed only once.

To ensure that the higher survival pattern found in CCR6 KO mice was related to the immunity against the malaria liver stage, we quantified the liver parasitic loads in the challenged mice through qPCR. After the exposure to either 250 live sporozoites via the intravenous route (A) or the biting of three infected mosquitoes (B), equivalent liver parasitic loads were detected in both mouse strains (p > 0.05 - Fig. 3A-B). Therefore, our data indicate that the global murine CCR6 expression is associated with a decreased malarial survival elicited by PbNK65 liver-stage parasites.

**Fig. 4:**
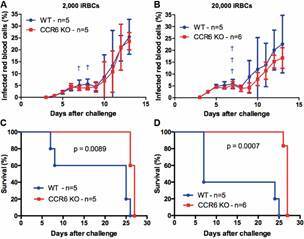
CCR6 KO mice presented a longer survival upon malaria induced by the intravenous injection of *Plasmodium berghei* NK65-infected red blood cells than wild-type (WT) mice. Mice were challenged with 2,000 (A) or 20,000 infected RBCs. (A-B) Parasitemia levels (mean ± standard deviation) were quantified from three days after the challenge onward in Giemsa stained-blood smears slides. † Number of mice found dead. (C-D) Kaplan-Meier curves represent the daily survival of challenged mice. Log-rank tests were performed to these analyses. (C) 2,000 or (D) 20,000 iRBCs). Circles - WT mice; Squares - CCR6 KO mice. Two independent experiments were performed for the 2,000 iRBC challenge, while the 20,000 iRBC challenge was run only once.

*CCR6 expression also decreases malaria survival in mice infected with PbNK65 blood-stage parasites* - Although liver parasite loads and parasitemia levels were undistinguishable between WT and CCR6 mice after sporozoite challenges, the latter animals still displayed enhanced survival against PbNK65 malaria (Figs 1-3). Thus, we investigated whether the CCR6 expression could disturb specifically the immunity against blood-stage parasites. For this, these mouse strains were intravenously (iv) infected with 2,000 or 20,000 PbNK65-infected red blood cells (iRBCs). In these challenges, both mouse strains showed similar parasitemia levels (Fig. 4A-B). Similar to the exposure to liver-stage parasites, CCR6 KO mice remained presenting a prolonged survival relative to WT mice, regardless the amount of iRBCs used (2,000 iRBCs - p = 0.0089; 20,000 iRBCs - p = 0.0007) (Fig. 4C-D). In conclusion, our data suggest that the global CCR6 expression can be detrimental to the murine survival against PbNK65 blood-stage parasites.

**Fig. 5:**
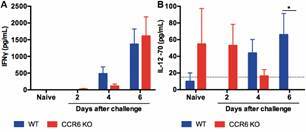
CCR6 KO mice secreted a lower production of IL-12 p70 after a malarial challenge in the serum than wild-type (WT) mice. Sera samples derived from infected WT (n = 7) and CCR6 KO (n = 9 for all time points, except day 6 that had n = 6) mice were collected at various time points for the measurement of IFN-γ (A) and IL-12 p70 (B) concentrations through enzyme-linked immunosorbent assay (ELISA). Naïve WT and CCR6 KO mice were utilized as negative controls (n = 6). Data represent the mean ± standard error of mean. Cytokine concentrations were compared through a two-independent sample student’s t-test in all-time points between both mouse strains. Blue columns - WT mice; Red columns - CCR6 KO mice. Dashed line - limit of detection; *p < 0.05. This experiment was performed only once.

*CCR6 KO mice have a reduced IL-12 p70 secretion in the serum upon the PbNK65-iRBC challenge in comparison to WT mice* - Pro-inflammatory cytokines, such as IL-12 p70 and IFN-γ, have been described as harmful factors for the malaria resistance induced by PbNK65-iRBCs in mice. Indeed, treatments with anti-IL-12 or anti-IFN-γ monoclonal antibodies strengthen the malaria survival as much as observed in PbNK65-iRBC challenged IL-12 KO or IFN-γ KO mice.^12^ ^,^ ^13^ Based on our previous data (Figs 1-4), we hypothesized that the enhanced malaria survival detected in CCR6 KO mice could be related to lower secretion levels of pro-inflammatory cytokines after the parasite exposure. Hence, IFN-γ and IL-12 p70 levels were quantified in serum samples of these mouse strains pre and post-challenge with PbNK65-iRBCs (days 2, 4, and 6) by ELISA. A comparable IFN-γ production was observed between WT and CCR6 KO mice throughout the time points evaluated (Fig. 5A). In contrast, the IL-12p70 production was significantly diminished to an undetectable level in CCR6 KO mice 6 days after infection when compared to WT mice (p < 0.05 - Fig. 5B). Therefore, our data propose that the prolonged survival of CCR6 KO mice after PbNK65 malaria is associated with a decreased serum IL-12 p70 production.

## DISCUSSION

In this study, we confirmed hallmarking aspects of the PbNK65-iRBC infection in C57BL/6 mice, such as high parasitemia and early mortality.^12^ ^,^ ^13^ In this model, the malarial pathogenesis seems to be more severe when pro-inflammatory cytokines, such as IL-12 and IFN-γ, are produced.^13^ ^,^ ^14^ Of note, the PbNK65 virulence was recently associated with a single polymorphism identified in the DNA binding domain of the parasitic APiAP2 transcription factor. The presence of a nucleotide (cytosine) at the position 5468 of the APiAP2 sequence would trigger a lethal iRBC infection with high parasitemia,^21^ as observed in our data (Fig. 4A-B). Some PbNK65 lines can also induce malaria-associated acute respiratory distress syndrome (MA-ARDS) in C57BL/6 mice.^24^ However, our iRBC stock is composed of the New York line that can accumulate in the lungs and fat tissues without developing MA-ARDS in these animals.^25^

In disagreement with our hypothesis that the global recruitment of CCR6-expressing cells was critical for the immunity against the malarial liver-stage, some *in vitro* approaches suggested that the sporozoite infection can activate, at least, a transient inflammatory response in hepatocyte cell lineages (Hepa 1-6, and HepG2 cells)^26^ and primary cells.^27^ More specifically, the CCL20 expression can be up-regulated in these hepatocytes few hours upon the sporozoite invasion.^26^ This process elicits the rupture of the hepatocyte membrane, releasing inflammation-mediating cytosolic factors.^27^

In our study, it is important to highlight that the sporozoite numbers chosen for the mouse challenges followed the same potency scale usually injected by mosquitoes in the host skin.^28^ ^,^ ^29^ Despite the lack of a linear correlation between the sporozoite loads and infection, mosquitoes harboring over 10,000 sporozoites in their salivary glands facilitate the malaria establishment.^30^ Indeed, an injection surpassing 25,000 sporozoites [Supplementary data (Fig. 1)] or the utilization of mosquitoes exceeding 10,000 sporozoites in their salivary glands [Supplementary data (Fig. 2A-B)] may have overloaded the immune system of CCR6 KO mice, hampering their ability to survive for a longer period than WT mice. The mechanisms related to the prolonged survival of CCR6 KO mice might be associated with distinct types of malarial pathogenesis. More specifically, several studies showed that animal deaths seen nearly day 25 post PbNK65-iRBC infection strongly correlate with an exacerbated liver inflammation.^9^ ^,^ ^12^ ^,^ ^13^ ^,^ ^14^ Although mouse deaths detected at day 10 remain elusive, this animal model of malaria was also described to induce inflammation of the cerebral microvasculature with cerebral edema, congestion, death of endothelial cells, parenchymal hemorrhage, proliferation of glia, accumulation of erythrocytes and leukocyte adhesion, of which can culminate with an earlier death.^31^

The infusion of a specific antibody to systemically neutralize chemokines or cytokines is an elegant approach to investigate their roles in a determined condition. To evaluate the relevance of the CCR6-CCL20 axis in the malarial immunity, we also treated WT mice with anti-CCL20 monoclonal antibodies before and during the blood-stage infection. However, the treatment did not have any effect on parasitemia and survival (data not shown). Initially, we suspected that the mAb amount used per mouse was not enough to neutralize the circulating chemokine. Some studies have demonstrated that similar mAb doses could neutralize the respective target *in vivo*.^12^ ^,^ ^32^ ^,^ ^33^ Although most of the chemokine or cytokine neutralization attempts were not effective against this malaria model of infection, the use of transgenic mice confirmed the importance of some molecules in the immunity against this disease though. More specifically, IL-12 KO, iNOS KO, IL-27R KO, IL-23 (p19)-KO, IL-17A KO, Perforin KO, and MyD88 KO mice displayed a lower malarial pathogenesis than WT mice after an iRBC challenge.^13^ ^,^ ^15^ ^,^ ^16^ ^,^ ^17^

Regarding the capacity of CCR6 KO mice in producing pro-inflammatory cytokines, it has been already described that their peritoneal macrophages secrete a reduced IL-12 p70 concentration after LPS stimulation relative to the WT mouse counterparts. This issue was further associated with the higher resistance of CCR6 KO mice to cecal ligation and puncture-derived sepsis^34^ and *Yersinia* oral infection.^35^ Also, IL-12 p70 was demonstrated to activate some lymphocytes, such as NK and NKT cells (DX5-expressing cells) that can kill hepatocytes.^13^ ^,^ ^14^ Similar numbers of NK1.1+ cells (NK or NKT cells) were enumerated in the peritoneum of naïve WT and CCR6 KO mice.^34^ However, malaria can significantly increase these cell subsets in the murine liver.^14^ If CCR6 KO mice present a diminished frequency of these cell subsets and an ensuing milder malaria than WT mice, it needs to be confirmed.

Although the CCR6 receptor binds to CCL20 with high affinity, it also possesses a low affinity to some antimicrobial peptides known as beta-defensins.^36^ These molecules are usually expressed in several types of epithelia, which can be colonized during an infection.^37^ Despite all uncertainty about their effective mechanisms, they represent an important innate component of the immune system against bacteria,^38^ fungi,^39^ viral,^40^ and parasite infections, such as *Cryptosporidium parvum*.^41^ These peptides can also attract cells, such as monocytes,^42^ macrophages,^43^ dendritic cells, and T cells,^36^ and act as an endogenous ligand for TLR4.^44^ However, the chemotaxis to dendritic cells and T cells seems controversial about the CCR6 dependence.^43^ Regarding the malarial infection, any correlation between beta-defensins and protection has never been outlined.

*In conclusion* - We found evidence that the CCR6 expression can influence the survival against the PbNK65 liver- and blood-stage malaria in C57Bl/6 mice. Moreover, this study reinforced the idea that the survival of these animals seems to be associated with a lower Th1 pattern of immune response upon the parasitic exposure as previously shown.^12^ ^,^ ^13^
