# Supplementary material for: CCR6 expression reduces mouse survival upon malarial challenge with Plasmodium berghei NK65 strain
Source: Mem Inst Oswaldo Cruz. 2022 Jun 17;117:e210287. doi: 10.1590/0074-02760210287 (PMC9208320; doi:10.1590/0074-02760210287)
Supplement: Supplementary file 1 [file 1678-8060-mioc-117-e210287-s.pdf]

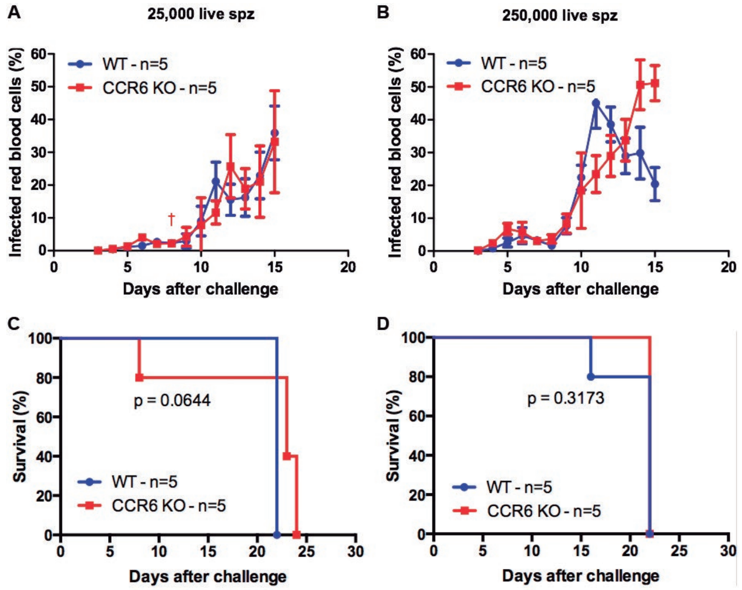

Fig. 1: extended survival of CCR6 KO mice upon a *Plasmodium berghei* NK65 sporozoite challenge is dependent on the inoculum size. Mice were infected via intravenous injection with 25,000 (A) or 250,000 (B) PbNK65 strain live sporozoites. (A-B) Parasitemia levels (mean ± standard deviation) were quantified three days after the challenge in Giemsa stained-blood smears slides (A) 25,000 or (B) 250,000 PbNK65 strain live sporozoites). † Number of mice found dead. C-D) Kaplan-Meier curves represent the daily survival of challenged mice (C) 25,000 or (D) 250,000 PbNK65 strain live sporozoites). Log-rank test was performed on this analysis. Circles - wild-type (WT) mice; Squares - CCR6 KO mice. This experiment was performed only once.

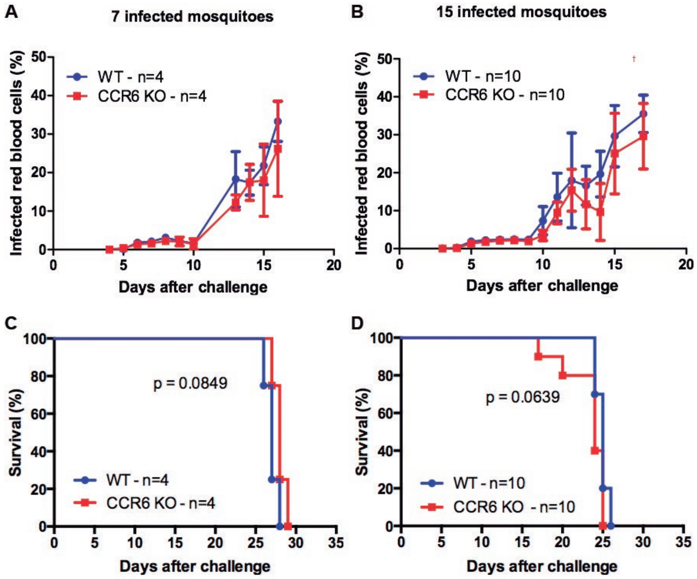

Fig. 2: extended survival of CCR6 KO mice exposed to the bites of *Plasmodium berghei* NK65-infected mosquitoes is dependent on the sporozoite inoculum size. Each mouse was anesthetized and exposed to the bites of seven (A) or 15 (B) infected mosquitoes for 15 min. About 10,000 (A) or 14,000 sporozoites (B) were enumerated in the salivary glands of each mosquito on the challenge day. (A-B) Parasitemia levels (mean ± standard deviation) were quantified three-four days after the challenge in Giemsa stained-blood smears slides (bites of seven (A) or 15 (B) infected mosquitoes). † Number of mice found dead. (C-D) Kaplan-Meier curves represent the daily survival of challenged mice (bites of seven (C) or 15 (D) infected mosquitoes). Log-rank test was performed on this analysis. Circles - wild-type (WT) mice; Squares - CCR6 KO mice. This experiment was performed only once.
